# Social Media Insights Into US Mental Health During the COVID-19 Pandemic: Longitudinal Analysis of Twitter Data

**DOI:** 10.2196/21418

**Published:** 2020-12-14

**Authors:** Danny Valdez, Marijn ten Thij, Krishna Bathina, Lauren A Rutter, Johan Bollen

**Affiliations:** 1 Department of Applied Health Science School of Public Health Indiana University Bloomington, IN United States; 2 Luddy School of Informatics, Computing, and Engineering Indiana University Bloomington, IN United States; 3 Psychological and Brain Sciences Indiana University Bloomington, IN United States

**Keywords:** social media, analytics, infodemiology, infoveillance, COVID-19, United States, mental health, informatics, sentiment analysis, Twitter

## Abstract

**Background:**

The COVID-19 pandemic led to unprecedented mitigation efforts that disrupted the daily lives of millions. Beyond the general health repercussions of the pandemic itself, these measures also present a challenge to the world’s mental health and health care systems. Considering that traditional survey methods are time-consuming and expensive, we need timely and proactive data sources to respond to the rapidly evolving effects of health policy on our population’s mental health. Many people in the United States now use social media platforms such as Twitter to express the most minute details of their daily lives and social relations. This behavior is expected to increase during the COVID-19 pandemic, rendering social media data a rich field to understand personal well-being.

**Objective:**

This study aims to answer three research questions: (1) What themes emerge from a corpus of US tweets about COVID-19? (2) To what extent did social media use increase during the onset of the COVID-19 pandemic? and (3) Does sentiment change in response to the COVID-19 pandemic?

**Methods:**

We analyzed 86,581,237 public domain English language US tweets collected from an open-access public repository in three steps. First, we characterized the evolution of hashtags over time using latent Dirichlet allocation (LDA) topic modeling. Second, we increased the granularity of this analysis by downloading Twitter timelines of a large cohort of individuals (n=354,738) in 20 major US cities to assess changes in social media use. Finally, using this timeline data, we examined collective shifts in public mood in relation to evolving pandemic news cycles by analyzing the average daily sentiment of all timeline tweets with the Valence Aware Dictionary and Sentiment Reasoner (VADER) tool.

**Results:**

LDA topics generated in the early months of the data set corresponded to major COVID-19–specific events. However, as state and municipal governments began issuing stay-at-home orders, latent themes shifted toward US-related lifestyle changes rather than global pandemic-related events. Social media volume also increased significantly, peaking during stay-at-home mandates. Finally, VADER sentiment analysis scores of user timelines were initially high and stable but decreased significantly, and continuously, by late March.

**Conclusions:**

Our findings underscore the negative effects of the pandemic on overall population sentiment. Increased use rates suggest that, for some, social media may be a coping mechanism to combat feelings of isolation related to long-term social distancing. However, in light of the documented negative effect of heavy social media use on mental health, social media may further exacerbate negative feelings in the long-term for many individuals. Thus, considering the overburdened US mental health care structure, these findings have important implications for ongoing mitigation efforts.

## Introduction

Beyond the obvious physical health ramifications of the COVID-19 pandemic, public health and the greater medical community is also bracing for a mental health crisis [1]. Within the span of 4 months, 45% of Americans indicated that the COVID-19 pandemic had taken a toll on their mental health, reporting higher levels of sadness and worsening of chronic psychiatric conditions [2]. Yet, despite an abundance of anecdotal evidence and peer-reviewed editorials that identify the potential mental health fallout of this public health crisis, the extent of these effects is empirically unknown.

Scientists mobilized quickly to measure many facets of the pandemic, including potential mental health effects. However, the time-consuming and costly nature of survey development [3] and instrument validation make it difficult to draw real-time conclusions [4], especially amid rapidly evolving news cycles that shift pandemic-related discourse. In the absence of survey data, social media represents a potentially valuable data source for studying emergent social issues, including the effect of those issues on behaviors and social mood [5]. Repeated tracking of social media data can provide a diachronic perspective on public morale and collective changes in sentiment, as participants voluntarily contribute to narratives, providing unprompted and diverse understandings of various issues [6-8]. Numerous scholars have successfully used social media data to identify trends and nuances in public mood using a combination of machine learning and artificial intelligence approaches. Some examples include comparing the happiness of users to their online social networks [9,10], identifying detailed predictors of mood through social media feeds [5], predicting cognitive distortions expressed among groups at risk of mental health disorders [11], tracking the emotions of social media users at high resolution [12,13], and mapping negative affectivity among users with internalizing disorders [14]. Collectively, these studies demonstrate the feasibility and value of using sentiment analysis on social media data to study societal mood and well-being, as well as biomedical signals among social media users that can provide useful proxies for mental health [12,15-17]. In fact, these approaches may be especially useful considering the speed that the pandemic became an acute socioeconomic phenomenon, the pervasiveness of COVID-19–related content available online, and the natural reaction of many to post on social media about pandemic-related events.

Indeed, throughout the COVID-19 pandemic, individuals have sought out crisis-related news at increased capacities [18], leading to a collective increase in global social media use [19]. This renders social media data about the COVID-19 pandemic a powerful source of information to draw real-time conclusions about aggregate social well-being during an unprecedented public health event. However, we must remember that, just as survey data are prone to biases, so are data derived from social media [20]. Therefore, to draw accurate inferences about mood, sentiment, and mental health, we must remain cognizant of the type of analysis performed, and what the analysis represents to measure nuanced aspects of sentiment. We contrasted topics discussed, topic-related sentiment, and personal sentiment to arrive at a more comprehensive and accurate assessment of changes in sentiments expressed through social media and their relevance to public health in the United States.

Broadly, this study answers the following three research questions (RQs):

- RQ1: What themes emerge from a corpus of US tweets about COVID-19?
- RQ2: To what extent did social media use increase during the onset of the COVID-19 pandemic?
- RQ3: What patterns emerge from longitudinal tracking of sentiment during the onset of the COVID-19 pandemic?

To address these RQs, we analyzed a large-scale set of Twitter data that are strictly relevant to the topic of COVID-19 in the United States from January 22, 2020, onward. Using this data, we also compiled a second corpus of individual geolocated social media timeline data from the same period to understand changes in personal sentiment as a proxy for mental health and evolving US perceptions of the COVID-19 pandemic.

## Methods

### Data

**Figure 1:**
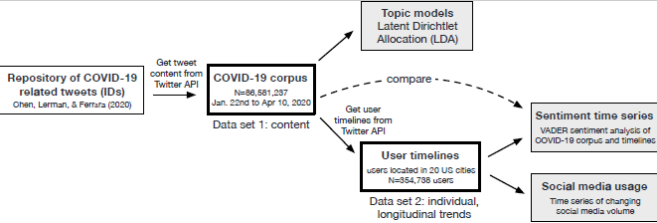
This diagram illustrates the procedure undertaken to procure Tweet IDs from an open-access COVID-19 repository. These tweet IDs were run through Twitter’s API to create two distinct data sets. The COVID-19 corpus, which contains all COVID-19–related English language tweets published in the United States between January-April 2020. The user timeline data contains the 3200 most recent tweets of users residing in the 20 cities most affected by COVID-19. API: application programming interface; VADER: Valence Aware Dictionary for Sentiment Reasoning.

We collected two distinct data sets, each reflecting different aspects of changes in social media behavior before and during the COVID-19 pandemic. The first data set of tweets, collected from an open-access repository containing all COVID-19–related tweets published in the United States [21], was designed to capture *topical differences* (ie, themes) in the Twitter discussion during the events that marked the onset of the pandemic. The repository provides a list of tweet IDs, which we used to extract tweet content from Twitter’s application programming interface (API; end point: GET statuses/show/id). We downloaded each tweet as well as the standard metadata provided by Twitter. Specifically, we retrieved COVID-19–related tweets posted between January 22, 2020 (first day of data collection and roughly 1 week prior to the first confirmed US COVID-19 case) through April 9, 2020 (the middle of social distancing efforts). Hereafter, we refer to this set of tweets as the “*COVID-19 corpus*” (n=86,581,237 tweets). Please refer to Figure 1 for a visual representation of this data set and how it was retrieved.

To gauge fluctuations of personal activity and mood at the individual (rather than topical) level, we downloaded the Twitter timelines (ie, the 3200 most recent tweets) of individual social media users who contributed to the COVID-19 corpus and resided within the 20 US cities with the most COVID-19 cases per 100,000 people from the Twitter API (end point: GET statuses/user_timeline). These timelines capture the changing behavior and emotions of individual Twitter users during the COVID-19 pandemic but do not strictly pertain to tweets exclusively related to COVID-19. We referred to this data as *user timeline data* (n=354,738 users; n=69,349,479 tweets), as shown in Figure 1. All tweets in either data set were scrubbed of any personally identifiable information in accordance with ethical social media use practices.

To ensure that we were measuring expressed sentiment in our data, we excluded non-English tweets and, specifically within the user timeline data, retweets and biasing keywords including “coronavirus,” “COVID-19,” and “pandemic,” among others. These words were removed because they inherently carry a negative connotation, and their inclusion would artificially decrease sentiment given that the corpus itself is composed of COVID-19–related content. In other words, because users are naturally tweeting about *coronavirus*, *virus*, and the *pandemic*, the inclusion of those words may not necessarily reflect the individual’s well-being. Note, the resulting sample sizes for each corpora exceeded the mean observed in a recent scoping survey of the literature on social media analytics for public health (n=20,000) [22], resulting in ample representation to conduct our analyses. Additionally, previous studies have used large-scale sentiment analysis to accurately predict social mood [23] and how sentiment expressed on social media correlates with psychological well-being [24]. Thus, the use of sentiment analysis for this study was appropriate.

### Analyses

#### Latent Dirichlet Allocation Topic Models

Latent Dirichlet allocation (LDA) topic models are unsupervised machine learning tools that perform probabilistic inferences to consolidate large volumes of text data into manageable themes [25]. Simply, words with high probabilities of association (ie, a high likelihood to appear in proximity with other words) are grouped together to form a latent theme, or topic, that qualitatively represents a content area within the collection of text. These methods have been applied in many ways such as to determine common themes in product reviews [26], to map themes within bodies of scientific literature [27], and to identify themes in social media data [28]. Thus, these tools are appropriate for exploratory analyses that seek to consolidate dense text data.

#### Sentiment Analysis

Sentiment analysis refers to a set of supervised or unsupervised machine learning and natural language processing techniques that extract affective or emotional indicators from text (eg, to determine whether a tweet expresses a negative or positive emotion about policy [23]). In this study, we used the Valence Aware Dictionary and Sentiment Reasoner (VADER) [29] to gauge the emotional valence of tweets. VADER is a rule-based open-source tool that recognizes common terms, idioms, abbreviations, and jargon while accounting for grammatical structures such as punctuation, negation, hedging, and magnification that are commonly employed in the vernacular of social media platforms. The VADER lexicon is one of the largest of its kind containing over 7500 common terms that are each rated for their emotional valence by 10 independent human raters. However, the word *virus* and its many variations (eg, viruses, viral) are not part of the VADER lexicon, meaning changes in the frequency of these words will not bias VADER scores. VADER has been extensively validated for Twitter content [30], showing some of the highest accuracy and coverage for tweets in a benchmark of more than 20 sentiment analysis tools [31].

#### Change-Point Detection

We applied the Pruned Exact Linear Time (PELT) change-point detection algorithm to identify significant changes in tweet volume and sentiment [32]. Change-point detection algorithms perform a set of mathematical operations over a time series (a series of time-based observations) to identify points in time where the statistical properties of the time series data changed significantly [33]. The PELT algorithm specifically attempts to find a set of change points for a given time series, such that their number and location in time minimizes a given segmentation cost. We chose the PELT algorithm over other similar change-detection algorithms because it is considered to be a more conservative estimate (preferring not to identify change points unless strict conditions are satisfied), thus yielding more accurate detection of statistical changes [34]. Additionally, PELT uses an offline approach to change detection [34], meaning it can consider all possible data points when identifying significant changes, regardless of the type of data, while maintaining high levels of performance.

### Procedure

#### What Themes Emerge From a Corpus of US Tweets About COVID-19?

We divided the COVID-19 corpus into daily segments and generated one topic model per day consisting of 20 topics each. We chose this number to reflect the widest possible span of themes while summarizing the major themes of the online discussion, which is a process used in previous studies [28,35]. We then looked at the top 20 associated words per topic and collapsed similar words into general themes, taking into account similarities of words (eg, United States and US) and potential misspellings, which are common in social media posts. As an example, *Hubei* and *Wuhan* were collapsed into the theme *China*. We then found the frequency ratio (ie, the number of occurrences of a certain word divided by the total number of words) of COVID-19–related themes (China, United States, pandemic, social distancing, Trump, home, lockdown, and deaths) and plotted them on a daily basis to show the evolution of topics over time, indicating both the contribution of the theme to all content of that day and the relative ranking of these terms among these themes. We used intercoder agreement methods to arrive at mutually agreeable interpretations of collapsed themes [36].

#### To What Extent Did Social Media Use Increase During the Onset of the COVID-19 Pandemic?

For this analysis we used the user timeline data instead of the general COVID-19 corpus (see Figure 1) because within-subject individual posting frequency is a better marker for tracking changes in social media use behavior [37]. Since Twitter’s API limits us to only the 3200 *most recent posting*s per individual, we only selected individuals who posted on Twitter before January 22, 2020, retaining 354,738 users. This ensured that our analytic sample captured individual behavior in the 20 most-affected US cities throughout the interval of interest (January 22, 2020, to April 9, 2020). We performed a seasonal decomposition—a method that separates the baseline, trend, and seasonal components of a time series—to determine whether we can observe increased Twitter use during the pandemic relative to events just prior. We then detected significant points of change using the PELT algorithm [38].

#### What Patterns Emerge From Longitudinal Tracking of Sentiment During the Onset of the COVID-19 Pandemic?

Sentiment can change because individuals discuss different topics (eg, using pejorative terms such as “virus” more frequently) or because of personal, individual changes in how people actually feel. We therefore compared daily VADER sentiment scores for the COVID-19 corpus (to gauge topical sentiment) and the user timeline within-subjects data (to assess personal changes in sentiment) from January 22, 2020 (the first official day of data collection) through April 9, 2020. We determined change points in the time series of daily averaged VADER sentiment with the PELT detection algorithm to identify significant changes in sentiment throughout this time period.

## Results

### What Themes Emerge From a Corpus of US Tweets About COVID-19?

We consolidated the COVID-19 corpus (n=86,581,237 tweets) into themes using LDA topic models. Figure 1 highlights the eight most salient topics and how their prominence (width of bars and rank) changed over time relative to major COVID-19–related milestones identified by the World Health Organization (WHO).

**Figure 2:**
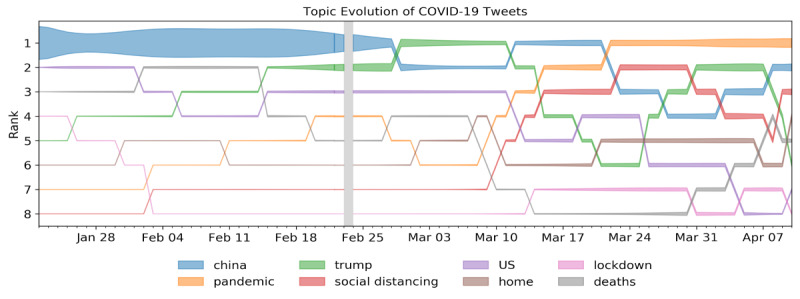
Topic group rankings over time in the COVID-19 corpus. Each topic is ranked by its frequency ratio. The width of the bars indicates the fraction of words in the topic on a given day. The colors of the areas indicate which theme the corresponding area belongs to.

As shown in Figure 2, topics continued to rise and fall in prominence relative to emerging news cycles throughout the time period under consideration. Indeed, the majority of COVID-19–related Twitter activity focused on China in February 2020. However, from March to April, as the novel coronavirus increasingly began to affect the US population, *China* became less prominent as more US-centered topics such as “lockdown” and “social distancing” emerged. Although China remained a prominent theme throughout the duration of interest, US-centered topics gradually came to dominate social media spaces.

### To What Extent Did Social Media Use Increase During the Onset of the COVID-19 Pandemic?

**Figure 3:**
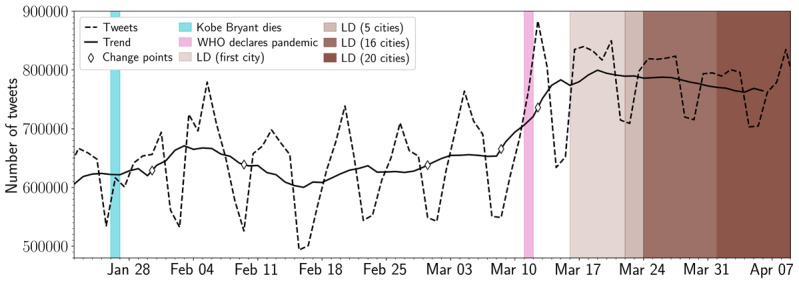
Number of daily tweets in a subsample of the user timeline data. The dashed line displays the daily number of tweets in the subsample of the user timeline data. The solid line indicates the underlying trend in the daily number of tweets (after removing its seasonal effects). The diamond markers indicate significant change points indicated by the Pruned Exact Linear Time algorithm. The light blue and pink annotations denote the death of Kobe Bryant (January 26, 2020) and the day when the WHO declared COVID-19 a global pandemic (March 11, 2020), respectively. The brown bars indicate the dates that lockdowns were enforced in the 20 considered cities (ranging from March 16 to April 1, 2020). The opacity of the brown bars indicates how many cities had enforced a lockdown at that date. LD: lockdown; WHO: World Health Organization.

Using user timeline data, we compared the frequency of social media posts before and during the pandemic of 292,000 users (whose timelines span January 22 onwards) in the 20 most-affected metropolitan cities (n= 66,725,505 tweets). Figure 3 highlights changes in posting volume between January 22, 2020, and April 9, 2020. The peaks and troughs (dashed line) of this graph show how seasonal and weekly cycles shape tweet volume. The solid line plots the trend in the time series after removing cycles and seasonal effects (through seasonal decomposition), and the progressively darker shades of brown denote the number of cities that imposed mandatory lockdowns.

Generally, we observed a consistent upward trend in total Twitter volume from early to late March. The PELT change-point algorithm identified two significant volume changes on March 8 and 12, 2020—around the time COVID-19 was declared a global pandemic (March 11) and President Trump declared a national emergency in the United States (March 14). The upward trend stabilized thereafter, albeit at higher observed volumes than prior to the onset of the COVID-19 pandemic. This supports the notion that individuals in our sample were more engaged with social media and made more use of it, possibly to discuss or obtain further information relevant to the news cycle.

### What Patterns Emerge From Longitudinal Tracking of Sentiment During the Onset of the COVID-19 Pandemic?

**Figure 4:**
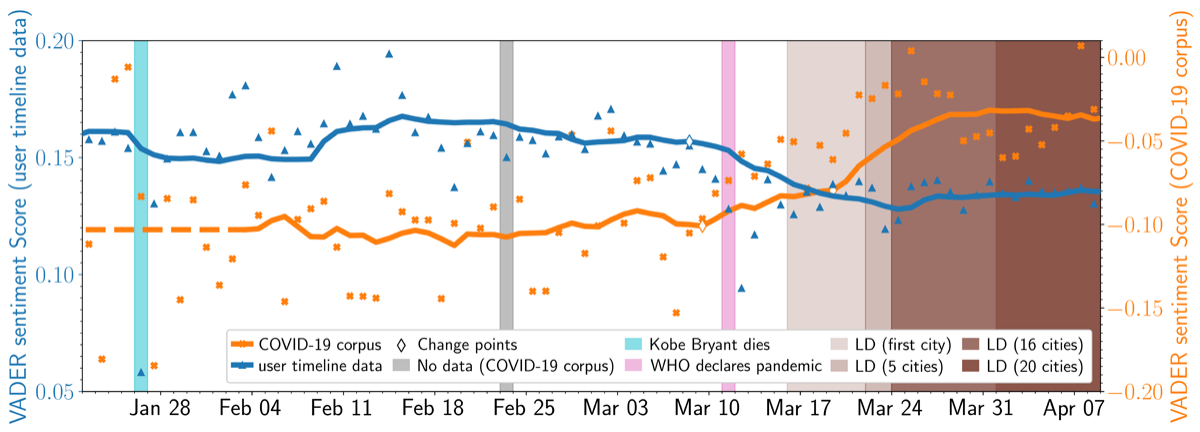
Distribution of nonzero VADER sentiment in the COVID-19 corpus (orange) and user-timeline data (blue). The solid lines display the 14-day moving average, and orange crosses/blue triangles represent the actual daily average VADER sentiment value. The diamond markers show change points indicated by the Pruned Exact Linear Time algorithm; the color of the diamond's edge refers to the time series that this change point belongs. The light blue, gray, and pink annotations denote the day of Kobe Bryant’s death (January 26, 2020), the day of missing data in the COVID-19 corpus (February 23), and the WHO declaration of COVID-19 as a global pandemic (March 11), respectively. The brown bars indicate the dates that lockdowns were enforced in the 20 considered cities (ranging from March 16 to April 1, 2020). The opacity of the brown bars indicates how many cities had enforced a lockdown at that date. LD: lockdown; VADER: Valence Aware Dictionary for Sentiment Reasoning; WHO: World Health Organization.

**Figure 5:**
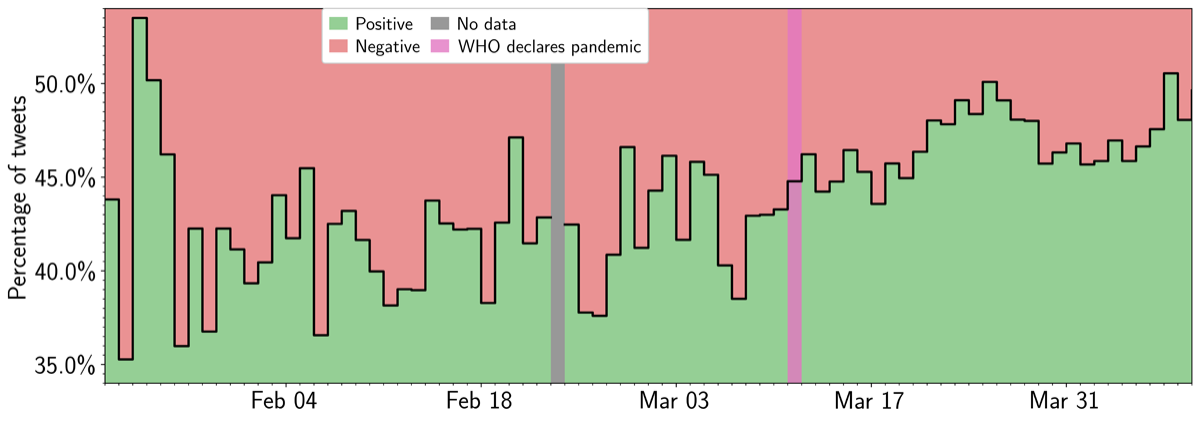
Distribution of positive and negative VADER sentiment in COVID-19 corpus. The solid line displays the fraction of tweets that hold positive or negative VADER sentiment in the user timeline data (colored green for positive and red for negative sentiment). The gray and pink annotations denote the day of missing data (February 23, 2020) and the WHO declaration of COVID-19 as a global pandemic (March 11), respectively. VADER: Valence Aware Dictionary for Sentiment Reasoning; WHO: World Health Organization.

We applied the VADER sentiment tool to the COVID-19 corpus (to assess sentiment of all US tweets about COVID-19) and the user timeline data (to track changes in user sentiment using their most recent 3200 tweets). Figure 4 tracks sentiment relative to major COVID-19–related milestones for both data sets, with the orange line tracking the COVID-19 corpus and the blue line tracking the user timeline data. In the COVID-19 corpus, there was an unmistakable increase in sentiment with two PELT-identified significant changes on March 9 (just before the WHO classified COVID-19 as a pandemic) and March 19, 2020 (shortly after President Donald Trump declared a national emergency). Figure 5 further shows that the percentage of positively scored COVID-19 tweets increased over time, reinforcing the positive trend.

Conversely, the user timeline data (which again contained the most recent 3200 tweets of a given user) showed decreases in sentiment over the same period. The user timeline data had one PELT-identified significant change in sentiment on January 28, 2020 (the day National Basketball Association [NBA] player Kobe Bryant died in a helicopter crash). There was a notable but short-lived drop in sentiment before March 9 (when the WHO classified COVID-19 as a pandemic).

## Discussion

### Principal Findings

The purpose of this study is to draw conclusions about US mental health amid the COVID-19 pandemic using computational social media analytics of a publicly available repository containing all COVID-19–related tweets. Using the COVID-19 tweet repository released by Chen and colleagues [21], we expanded on their original topical analysis of COVID-19 content by examining both topics and sentiment specific to the US population to understand how the COVID-19 pandemic may be impacting social well-being. We used social media data as the medium for analysis for several reasons, in particular its usefulness to gauge real-time changes in mental health and social well-being before, during, and after rapid socioeconomic changes [15]. In this section, we discuss the findings of this study in detail and highlight public health implications about social well-being during the onset of the COVID-19 pandemic.

### COVID-19 Social Media Themes: Content Is Reactionary to News Cycles

Bento and colleagues [18] predicted that crisis-related information seeking would increase during the COVID-19 pandemic. Numerous studies have supported that prediction by identifying increased panic within social media spaces as users react to COVID-19 news–related content on their feeds [39,40]. Through our LDA topic modeling analysis, we echo much of those findings as well; the topics uncovered by LDA frequently corresponded to the life cycles of COVID-19–related news and current events. For example, the name used to identify the virus on social media evolved to match changes in viral terminology as presented on news outlets (eg, the *novel coronavirus* became *coronavirus*, *COVID-19*, and *COVID-19 pandemic*). The locations that emerged within the topics also corresponded to the movement of COVID-19 from mainland China to the United States. For example, in late January, *China* was a dominant theme in Twitter content (shown by the width of the bars in Figure 2), indicating the US public may have considered the then-classified epidemic a foreign matter (eg, tweet: “OMG China just shut down trading...Still think it's just a flu?”). However, in later weeks and months, US-centered topics and tweets replaced *China* in prominence as more Americans became preoccupied with pandemic control measures at home (eg, tweet: “lol it’s wild seeing every single other country grappling with this virus in ways we KNOW the US will never do”). These findings suggest that, as the COVID-19 pandemic became more prominent in the United States, social media content changed to topics of more direct impact to the United States and with greater frequency of use (evidenced by a PELT-identified significant increase in Twitter volume during that time). This volume increase remained consistently above levels observed prior to the COVID-19 pandemic, which is possibly explained by several factors including increased anxiety as the COVID-19 pandemic reached the United States, the isolating effects of statewide stay-at-home orders, or other social fallouts driven by the pandemic. These findings collectively support those of Castillo and colleagues [41], who contend that social media content follows the life cycle of news stories. Thus, news, as a vehicle for crisis-related social media communication, should be studied more intently.

### COVID-19 Topical Sentiment: Increased Sentiment May Indicate a Priming Effect

Given the expected negative physical and emotional outcomes of a major pandemic, we were surprised to observe that the sentiment of the COVID-19 corpus trended positively. However, this increase in sentiment is likely not indicative of actual changes in population sentiment but rather the effect of a common *topical* bias in social media analytics [42]. Our COVID-19 data set was specifically selected to contain tweets that related to the topic of COVID-19. Hence, its sentiment scores will reflect the language used to discuss this particular topic and not necessarily the underlying emotion of the population. Topically selected social media posts are likely primed by news cycles [43] and show false optimism—a phenomena where individuals tend to post content that is more positive or optimistic than their true emotions [44]. Previous research has further shown that language in general is biased toward positivity, especially when posts are collected for a random topic [30]. For example, language used to compose a post about COVID-19 may contain trending verbiage or framing devices, including showcasing support of groups frequently part of news cycles (eg, tweet: “Great news...it’s a welcome burden lifted off our incredible nurses and medical first responders”). Similarly, users may just be conveying positivity through carefully selected *popular* words around a trending topic (eg, tweet: “No matter how hard the situation nowadays during the pandemic outbreak, we should keep being positive and optimistic”). Within the VADER lexicon, many of the words commonly used in this context are scored positively (great, welcome, incredible, positive, optimistic), which also artificially inflates sentiment ratings. Thus, we posit that topically driven tweet samples may not validly reflect actual changes in population mood but rather topic-driven language sentiment. This justifies our approach to analyze within-subject timelines of individual posts that are not necessarily bound by the criterion of strictly being COVID-19–related, thereby increasing the odds of reflecting personal changes in mood trajectories.

### User Timeline Sentiment: Lower Timeline Sentiment May Indicate Decreased Social Well-being

By contrast, we found a negative trajectory in sentiment scores for the user timeline data. This means that, although content in the COVID-19 corpus trended positively (possibly due to priming), relative to the totality of their timelines, our sample mood was *lower* than it once was. Thus, this comparison gives us deeper insight into *underlying* mood and sentiment. This finding further supports the assertion that the positivity conveyed in social media posts may not validly reflect what people are feeling at a given moment. Indeed, that positivity may be serving as a veneer posted *in the moment* to convey positivity during a time of uncertainty. To obtain an accurate assessment of mood and well-being, other reference points (in this case, social media posts prior to the pandemic) are needed to examine in-the-moment sentiment relative to their prior histories. Within our timeline data, we captured one event that occurred prior to the pandemic that also affected sentiment scores relative to timeline histories—the passing of NBA basketball player Kobe Bryant. The effect of Bryant’s passing led to a sharp decline in sentiment, which lasted approximately 24 hours before returning to levels observed previously. Regarding the pandemic, after the PELT-identified shift on March 8, 2020, sentiment scores (relative to timelines) became not only lower but also consistently lower and did not return to levels observed before the pandemic reached the United States. This trend may hold implications into the longitudinal effects of the pandemic and subsequent impacts on US mood and social sentiment, as this continued trend may be indicative of a long-lasting shift in mood and well-being.

### Social Media Use in Times of Crisis

Analyses of aggregated social media feeds are shown to adequately predict other phenomena including the stock market [45]; political leanings [46]; and, when analyzed through a time series, collective shifts in general mood [47]. Our study contributes to this body of literature by highlighting the disparity between how Americans portray themselves on social media versus the latent sentiment they may be experiencing during times of crisis. Generally, Americans were not posting social media content about COVID-19 prior to the first documented US case. Once COVID-19 became a reality in the United States, however, there was a continued increase in the total number of US-based tweets about the novel coronavirus, indicative of growing social media use in our sample. By analyzing the COVID-19 corpus and user timeline data separately, but with the same analytic procedures, we saw divergent findings that reinforced the difference between in-the-moment portrayals versus the longitudinal information that can be gleaned from individual timeline analyses. For example, tweets and posts about COVID-19 may attempt to be lighthearted or convey optimism; however, individually, social media users may not be as optimistic as they were prior to the pandemic. During an unprecedented public health crisis, it is therefore important to look beyond the topical focus of messages on social media that reference the crisis itself as a proxy of public mood, as they are likely to be affected by other influences (eg, political framing and projecting hope). Ultimately, our findings exemplify COVID-19 as a case study in social media behavior, whose outcomes should be generalized to other crisis-related events.

### Concluding Remarks and Implications

At the time of writing, the US COVID-19 death toll was just over 136,000, with the lives of millions of people disrupted by the various effects of the pandemic. This study elucidates the possible mental health effects of the COVID-19 pandemic among Twitter users using a computational approach to analyze a corpus of all archived US-based COVID-19 tweets from January to April 2020. These analyses revealed, to varying extents, how the pervasiveness of COVID-19 content available on social media and abrupt shift in lifestyles may be negatively affecting social sentiment relative to points just prior to the pandemic. Given that sentiments expressed on social media have been used as a proxy for mental well-being [48], these findings support calls from public health and medical scholars who contend that mental health is an urgent concern during the COVID-19 pandemic, especially as our findings illustrate a declining trend in sentiment. Thus, we encourage further research on US mental health status amid the pandemic using survey methods or other primary data collections to substantiate our findings with testable outcomes. We also call for more research on mental health interventions amid the COVID-19 pandemic, with particular attention to modality (ie, in person vs virtual), and the efficacy of those efforts.

### Limitations

Our study is subject to limitations. Twitter requires users to opt in to geotagging features. Consequently, any information inferred about the user’s city of residence is often limited to self-reported data as specified on profile pages. This means that some of our timeline data may not originate from the city specified by a user, as this information can easily be misrepresented [49] (eg, stating they live in New York, NY, but actually residing in Newark, NJ). We also acknowledge a likely bias regarding key demographic information, including age, gender, and socioeconomic status among social media users [50], in addition to the temporal, spatial, and geographic patterns that may affect how sentiment is expressed on social media (eg, older adults posting early in the morning vs younger adults posting late at night or urban vs rural users) [51]. It is also, as of yet, not possible to accurately diagnose someone with a mental health condition through social media feeds alone, although research has shown that social media content contains important indicators with respect to mental health and biomedical signals. Thus, we relied on trend data to draw inferences about the *possibility* of mental health decline based on average sentiment scores [52]. However, these limitations do not diminish the importance or validity of this study. Rather, they create avenues for additional research that expand on the findings of this paper and leverage the limitations inherent to social media data, such as measuring cognitive distortions on Twitter based on posting time or approaches that measure diagnosable mental health conditions through social media data, particularly during times of increased panic and crisis. In addition, because social media has been widely used to draw conclusions about public mood through large-scale sentiment analysis procedures [10,18], we contend our approach is appropriate to draw the conclusions discussed herein. See Multimedia Appendix 1 for source code.

## Appendix

Multimedia Appendix 1 Study syntax.

## Abbreviations

API: application programming interface
LDA: latent Dirichlet allocation
NBA: National Basketball Association
PELT: Pruned Exact Linear Time
RQ: research question
VADER: Valence Aware Dictionary and Sentiment Reasoner
WHO: World Health Organization

## References

[1] Auerbach J, Miller BF (2020). COVID-19 exposes the cracks in our already fragile mental health system. Am J Public Health.

[2] Pfefferbaum B, North CS (2020). Mental health and the covid-19 Pandemic. N Engl J Med.

[3] Gualano MR, Lo Moro G, Voglino G, Bert F, Siliquini R (2020). Effects of covid-19 lockdown on mental health and sleep disturbances in Italy. Int J Environ Res Public Health.

[4] Coughlan M, Cronin P, Ryan F (2009). Survey research: process and limitations. Int J Ther Rehabil.

[5] Berry N, Emsley R, Lobban F, Bucci S (2018). Social media and its relationship with mood, self-esteem and paranoia in psychosis. Acta Psychiatr Scand.

[6] Cho SE, Jung K, Park HW (2013). Social media use during Japan's 2011 earthquake: how Twitter transforms the locus of crisis communication. Media Int Aust.

[7] Page RE (2013). Stories and Social Media: Identities and Interaction.

[8] Stieglitz S, Dang-Xuan L (2014). Emotions and information diffusion in social media—sentiment of microblogs and sharing behavior. J Manage Inf Syst.

[9] Bollen J, Gonçalves B, Ruan G, Mao H (2011). Happiness is assortative in online social networks. Artif Life.

[10] Bollen J, Gonçalves B, van de Leemput I, Ruan G (2017). The happiness paradox: your friends are happier than you. EPJ Data Sci.

[11] Simms T, Ramstedt C, Rich M, Richards M, Martinez T, Giraud-Carrier C (2017). Detecting cognitive distortions through machine learning text analytics.

[12] Correia RB, Wood IB, Bollen J, Rocha LM (2020). Mining social media data for biomedical signals and health-related behavior. Annu Rev Biomed Data Sci.

[13] Golder SA, Macy MW (2011). Diurnal and seasonal mood vary with work, sleep, and daylength across diverse cultures. Science.

[14] Johns Hopkins University Bloomberg School of Public Health (2019). Social media use by adolescents linked to internalizing behaviors. Medical Xpress.

[15] Bollen J, Pepe A, Mao H (2009). Modeling public mood and emotion: Twitter sentiment and socio-economic phenomena. arXiv.

[16] Dodds PS, Clark EM, Desu S, Frank MR, Reagan AJ, Williams JR, Mitchell L, Harris KD, Kloumann IM, Bagrow JP, Megerdoomian K, McMahon MT, Tivnan BF, Danforth CM (2015). Reply to Garcia et al.: common mistakes in measuring frequency-dependent word characteristics. Proc Natl Acad Sci U S A.

[17] Bekalu MA, McCloud RF, Viswanath K (2019). Association of social media use with social well-being, positive mental health, and self-rated health: disentangling routine use from emotional connection to use. Health Educ Behav.

[18] Bento AI, Nguyen T, Wing C, Lozano-Rojas F, Ahn YY, Simon K (2020). Information seeking responses to news of local COVID-19 cases: evidence from internet search data. arXiv. Preprint posted online April 6,.

[19] Koeze E, Popper N (2020). The virus changed the way we internet. The New York Times.

[20] Olteanu A, Castillo C, Diaz F, Kıcıman E (2019). Social data: biases, methodological pitfalls, and ethical boundaries. Front Big Data.

[21] Chen E, Lerman K, Ferrara E (2020). COVID-19: the first public coronavirus Twitter dataset. Preprint posted online March 16,.

[22] Edo-Osagie O, De La Iglesia B, Lake I, Edeghere O (2020). A scoping review of the use of Twitter for public health research. Comput Biol Med.

[23] Liu B (2012). Sentiment analysis and opinion mining. Synthesis Lect Hum Lang Technologies.

[24] Jaidka K, Giorgi S, Schwartz HA, Kern ML, Ungar LH, Eichstaedt JC (2020). Estimating geographic subjective well-being from Twitter: a comparison of dictionary and data-driven language methods. Proc Natl Acad Sci U S A.

[25] Blei DM, Ng AY, Jordan MI (2003). Latent Dirichlet allocation. J Machine Learning Res.

[26] Wang H, Ding Y, Tang J, Dong X, He B, Qiu J, Wild DJ (2011). Finding complex biological relationships in recent PubMed articles using Bio-LDA. PLoS One.

[27] Moghaddam S, Ester M (2011). ILDA: interdependent LDA model for learning latent aspects and their ratings from online product reviews. Proceedings of the 34th international ACM SIGIR Conference on Research and Development in Information Retrieval.

[28] Barry AE, Valdez D, Padon AA, Russell AM (2018). Alcohol advertising on Twitter—a topic model. Am J Health Education.

[29] Hutto CJ, Gilbert E (2014). VADER: a parsimonious rule-based model for sentiment analysis of social media text.

[30] Elbagir S, Yang J (2019). Twitter sentiment analysis using natural language toolkit and VADER sentiment. Proceedings of the International MultiConference of Engineers and Computer Scientists.

[31] Ribeiro MT, Singh S, Guestrin C (2016). Model-agnostic interpretability of machine learning. arXiv.

[32] Killick R, Fearnhead P, Eckley IA (2012). Optimal detection of changepoints with a linear computational cost. J Am Stat Assoc.

[33] Liu S, Yamada M, Collier N, Sugiyama M (2013). Change-point detection in time-series data by relative density-ratio estimation. Neural Netw.

[34] Wambui GD, Waititu GA, Wanjoya A (2015). The power of the Pruned Exact Linear Time(PELT) test in multiple changepoint detection. Am J Theor Appl Stat.

[35] Valdez D, Pickett AC, Goodson P (2018). Topic modeling: latent semantic analysis for the social sciences. Soc Sci Q.

[36] Kuckartz U, Rädiker S (2019). Analyzing intercoder agreement. Analyzing Qualitative Data with MAXQDA: Text, Audio, and Video.

[37] Lee K, Agrawal A, Choudhary A (2013). Real-time disease surveillance using Twitter data: demonstration on flu and cancer. Proceedings of the 19th ACM SIGKDD International Conference on Knowledge Discovery and Data Mining.

[38] Gabrielli L, Rinzivillo S, Ronzano F, Villatoro D, Nin J, Villatoro D (2014). From tweets to semantic trajectories: mining anomalous urban mobility patterns. Citizen in Sensor Networks.

[39] Ahmad AR, Murad HR (2020). The impact of social media on panic during the COVID-19 pandemic in Iraqi Kurdistan: online questionnaire study. J Med Internet Res.

[40] Depoux A, Martin S, Karafillakis E, Preet R, Wilder-Smith A, Larson H (2020). The pandemic of social media panic travels faster than the COVID-19 outbreak. J Travel Med.

[41] Castillo C, El-Haddad M, Pfeffer J, Stempeck M (2014). Characterizing the life cycle of online news stories using social media reactions. Proceedings of the 17th ACM Conference on Computer Supported Cooperative Work and Social Computing.

[42] Ferrara E, Yang Z (2015). Measuring emotional contagion in social media. PLoS One.

[43] Schleuder JD, White AV, Cameron GT (1993). Priming effects of television news bumpers and teasers on attention and memory. J Broadcasting Electronic Media.

[44] Bala K (2014). Social media and changing communication patterns. Global Media J-Indian Edition.

[45] Bollen J, Mao H, Zeng X (2011). Twitter mood predicts the stock market. J Computational Sci.

[46] Makazhanov A, Rafiei D, Waqar M (2014). Predicting political preference of Twitter users. Soc Network Analysis Mining.

[47] Coppersmith G, Dredze M, Harman C, Hollingshead K (2015). From ADHD to SAD: analyzing the language of mental health on Twitter through self-reported diagnoses.

[48] Derks D, Fischer AH, Bos AE (2008). The role of emotion in computer-mediated communication: a review. Comput Hum Behav.

[49] Gore RJ, Diallo S, Padilla J (2015). You are what you tweet: connecting the geographic variation in America's obesity rate to Twitter content. PLoS One.

[50] Smith A, Brenner J (2012). Twitter Use 2012. Pew Research Center.

[51] Padilla JJ, Kavak H, Lynch CJ, Gore RJ, Diallo SY (2018). Temporal and spatiotemporal investigation of tourist attraction visit sentiment on Twitter. PLoS One.

[52] De Choudhury M, De S (2014). Mental health discourse on Reddit: self-disclosure, social support, and anonymity.

