# Supplementary material for: Social Media Insights Into US Mental Health During the COVID-19 Pandemic: Longitudinal Analysis of Twitter Data
Source: J Med Internet Res. 2020 Dec 14;22(12):e21418. doi: 10.2196/21418 (PMC7744146; doi:10.2196/21418)
Supplement: Multimedia Appendix 1 [file jmir_v22i12e21418_app1.docx]

APPENDIX: STUDY SYNTAX

**RQ1:**

import matplotlib

import matplotlib.pyplot as plt

import glob

import pandas as pd

import numpy as np

import re

import gzip as gz

from collections import Counter

from collections import defaultdict

from scipy.special import comb

from scipy import stats

import matplotlib.ticker as ticker

from matplotlib.ticker import (MultipleLocator, FormatStrFormatter,

                               AutoMinorLocator)

from sklearn.cluster import KMeans

from multiprocessing import Pool

from matplotlib.dates import WeekdayLocator, DayLocator, DateFormatter

## estimating the flow by fitting a curve

def bernstein_poly(i, n, t):

    """

     The Bernstein polynomial of n, i as a function of t

    """

    return comb(n, i) * ( t**(n-i) ) * (1 - t)**i

def bezier_curve(points, nTimes=1000):

    nPoints = len(points)

    xPoints = np.array([p[0] for p in points])

    yPoints = np.array([p[1] for p in points])

    t = np.linspace(0.0, 1.0, nTimes)

    polynomial_array = np.array([ bernstein_poly(i, nPoints-1, t) for i in range(0, nPoints)   ])

    xvals = np.dot(xPoints, polynomial_array)

    yvals = np.dot(yPoints, polynomial_array)

    return xvals, yvals

# replacing similar words to a common word

to_replace = {'(?i)(.*)corona(.*)': 'corona',

              '(?i)covid(.*)': 'corona',

              '(?i)ncov(.*)': 'corona',

              '(?i)virus(.*)': 'corona',

              '(?i)chin(.*)': 'china',

              '(?i)wuhan(.*)': 'china',

              '(?i)hubei(.*)': 'china',

              '(?i)province(.*)': 'china',

              '(?i)pence(.*)': 'trump',

              '(?i)(.*)donald(.*)': 'trump',

              '(?i)case(?i)':'cases',

              '(?i)distancing': 'SD',

  '(?i)social: 'SD',

              '(?i)(.*)american(.*)':'US',

              '(?i)(.*)american(.*)':'US',

              '(?i)(.*)death(.*)':'deaths',

              '#(.*)':'',

             '^[-+]?[0-9]+$':'',

             '“':'','”':'','\...':'',

             'us':'US'}

def replace(token):

    for word,rep in to_replace.items():

        token = re.sub(word, rep, token)

    return token

# read in LDA data

# list of top 20 words in each topic per day

topics = defaultdict(list)

with open('LDA/LDA.csv') as f:

    data = [x.strip().split(',') for x in f.readlines()]

for d in data:

    topics[d[0]].extend([replace(word) for word in d[2:]])

cleaned = {}

# for each day, get the ratio of word usage

for d,values in topics.items():

    c = Counter(values) # get a counter

    s = sum(c.values()) # get total sum of counter

    c = c.most_common() # order counter by frequency

    cleaned[d] = {x:y/s for x,y in c if len(x) > 1} # calculate ratio

## convert to dataframe

## replace SD to social distancing (for graph label)

df = pd.DataFrame(cleaned).fillna(0).T

df.index = pd.to_datetime(df.index)

df.rename({'SD': 'social distancing'}, axis=1, inplace = True)

df = df[df.index < '2020-04-11'] # filter data to april 10

def create_data(words, init, spacing = 0.005):

    '''

    calculate the top and bottom of the flow for each word

    '''

    t = df[words].T # subset data

    tops = {date:{x:None for x in words} for date in t.columns} # initialize dictionary to store location of words in graph

    bottoms = {date:{x:None for x in words} for date in t.columns} # initialize dictionary to store location of words in graph

    spacing = spacing # set a spacing value between flows

    for date in t.columns: # for each day

        inds = t[date].rank(method='min').sort_values().index # get a rank ordering of words

        areas = t[date].sort_values().values # get an ordering of the frequency ratio

        bottoms[date][inds[0]] = init # for the plot, the bottom value of the first word is the initial starting point

        tops[date][inds[0]] = init+areas[0] # for the plot, the top value of the first word is the initial point plus its ratio

        for i in range(1,len(inds)): # for the rest of the words, update the same way but add a spacing in between each one

            bottoms[date][inds[i]] = tops[date][inds[i-1]] + spacing

            tops[date][inds[i]] = bottoms[date][inds[i]] + areas[i]

    return t, bottoms,tops

# choose words of interest for LDA

words = ['china', 'pandemic', 'trump','social distancing','US','home','lockdown','deaths']

t,bottoms,tops = create_data(words, init = 0, spacing = 0.005) # get placements for flow diagram

# store the hashtag values in an array

hashtag_ratios = np.array(list(maxs_dict.values()))

# add hashtag symbol

word_labels = ['#' + x for x in np.array(list(maxs_dict.keys()))]

# replace character with a dash - the original dash character can't be viewed on matplotlib

word_labels = [x if x != '#covidー19' else '#covid-19' for x in word_labels]

l = []

for word in t.index:

    points = list(zip(range(len(t.columns)), t.loc[word].values))

    xbez, ybez = bezier_curve(points, nTimes=79 * 15)

    l.append(ybez[::-1])

temp = pd.DataFrame(l)

temp.index = t.index

temp = temp.T.groupby(pd.qcut(temp.T.index,79)).mean()

temp.index = t.columns

temp = temp.T

temp.columns = pd.to_datetime(temp.columns)

temp[pd.Timestamp('2020-02-23 00:00:00')] = np.nan

data = temp[sorted(temp.columns)]

ranks = data.rank(ascending=False, method="dense")

fig = plt.figure(figsize=(13, 4))

ax = fig.add_subplot(111)

for x,word in enumerate(words):

    ax.fill_between(data.columns, ranks.loc[word, :] - 4 * data.loc[word, :], ranks.loc[word, :] + 4* data.loc[word, :],

                    color="C{}".format(x), lw=1, alpha=0.5, zorder=2, label = word)

    ax.fill_between([pd.Timestamp('2020-02-22'), pd.Timestamp('2020-02-23')],

                    [ranks.loc[word, '2020-02-22'] - 4 * data.loc[word, '2020-02-22'],

                    ranks.loc[word, '2020-02-22'] - 4 * data.loc[word, '2020-02-24']],

                    [ranks.loc[word, '2020-02-22'] + 4* data.loc[word, '2020-02-22'],

                    ranks.loc[word, '2020-02-22'] + 4 * data.loc[word, '2020-02-24']],

                    color="C{}".format(x), lw=1, alpha=0.5, zorder=2)

legend = ax.legend(loc='lower center', bbox_to_anchor=(0.5, -0.35),

                   fontsize = 12, ncol = 4, frameon = False)

ax.set_xlim(pd.Timestamp("2020-01-22"), pd.Timestamp("2020-04-10"))

ax.xaxis.set_major_locator(WeekdayLocator())

ax.xaxis.set_minor_locator(DayLocator())

ax.xaxis.set_major_formatter(DateFormatter('%b %d'))

ax.set_ylabel("Rank", fontsize=12)

ax.set_ylim(0.1,8.5)

ax.yaxis.set_major_locator(matplotlib.ticker.MultipleLocator(1))

ax.invert_yaxis()

# ax.grid(which="minor", b=True, alpha=0.3, zorder=1)

# ax.grid(b=True, zorder=1)

ax.tick_params(labelsize=12)

ax.set_title('Topic Evolution of COVID-19 Tweets', fontsize = 12);

plt.axvspan('2020-02-23', '2020-02-24', facecolor="0.7", alpha=0.5, label="No data")

plt.savefig('Topic_evolution.png', dpi=300,

    bbox_inches='tight',bbox_extra_artists=[legend])

**RQ2, RQ3:**

import matplotlib as mpl

import matplotlib.dates as mdates

import matplotlib.pyplot as plt

import matplotlib.ticker as mticker

​

import pandas as pd

​

mpl.rcParams['figure.figsize'] = (16, 4)

mpl.rcParams['text.usetex'] = True

mpl.rcParams['text.latex.preamble'] = [r'\usepackage{amsmath,amssymb,amsthm,bbm}']

​

tab10 = plt.get_cmap('tab10').colors

fontsize = 28

labelsize = 22

tiny_labelsize = 16

ms = 6

​

_CITY_COLOR = tab10[0]

_COVID_COLOR = tab10[1]

_NEG_COLOR = tab10[3]

_POS_COLOR = tab10[2]

_ND_COLOR = tab10[7]

_KOBE_COLOR = tab10[9]

_WHO_COLOR = tab10[6]

_LD_COLOR = tab10[5]

​

_WHO_LABEL = r"WHO declares pandemic"

_KOBE_LABEL = r"Kobe Bryant dies"

​

_START = "2020-01-01"

_MISSING_DATE = "2020-02-23"

_START_COVID = "2020-01-22"

_END = "2020-04-09"

_WHO = "2020-03-11"

_KOBE = "2020-01-26"

_DAY = pd.Timedelta("1 day")

​

_FIG_FLDR = "figures/"

_DATA_FLDR = "results/"

​

_PD = pd.read_csv(_DATA_FLDR + "public_health_paper_plot_data.tsv", sep="\t", parse_dates=True, index_col=0)

​

fig = plt.figure(figsize=(16, 6))

ax = fig.add_subplot(111)

​

ax.plot(_PD.index, _PD["COVID_tweet_quantity"].values, lw=2, color="k", linestyle="--", label=r"Tweets")

ax.plot(_PD.index - pd.Timedelta(hours=12), _PD["COVID_tweet_trend"], lw=2, color="k", label=r"Trend")

ax.scatter(_PD.index, _PD["COVID_tweet_CP"], color="k", s=2 * ms ** 2, marker="d",

        edgecolor="k", facecolor="w", label=r"Change-points", zorder=3)

​

ax.set_title("Activity for a subsample of the user-timeline data (20 Major US cities)",

          fontsize=labelsize)

​

ax.fill_between([pd.Timestamp(_KOBE), pd.Timestamp(_KOBE) + _DAY], 480000, 900000,

             color=_KOBE_COLOR, alpha=0.5, label=_KOBE_LABEL, zorder=1)

ax.fill_between([pd.Timestamp(_WHO), pd.Timestamp(_WHO) + _DAY], 480000, 900000,

             color=_WHO_COLOR, alpha=0.5, label=_WHO_LABEL, zorder=1)

ax.fill_between([pd.Timestamp("2020-03-16"), pd.Timestamp("2020-03-22")], 480000, 900000,

             color=_LD_COLOR, alpha=0.24, label=r"LD (NOLA)", zorder=1)

ax.fill_between([pd.Timestamp("2020-03-22"), pd.Timestamp("2020-03-24")], 480000, 900000,

             color=_LD_COLOR, alpha=0.4, label=r"LD (5 cities)", zorder=1)

ax.fill_between([pd.Timestamp("2020-03-24"), pd.Timestamp("2020-04-01")], 480000, 900000,

             color=_LD_COLOR, alpha=0.84, label=r"LD (16 cities)", zorder=1)

ax.fill_between([pd.Timestamp("2020-04-01"), pd.Timestamp(_END)], 480000, 900000,

             color=_LD_COLOR, alpha=1, label=r"LD (20 cities)", zorder=1)

​

ax.set_xlim(pd.Timestamp(_START_COVID), pd.Timestamp(_END))

ax.xaxis.set_major_locator(mdates.WeekdayLocator())

ax.xaxis.set_minor_locator(mdates.DayLocator())

ax.xaxis.set_major_formatter(mdates.DateFormatter("%b %d"))

​

ax.set_ylabel(r"Number of tweets", fontsize=labelsize)

ax.set_ylim(480000, 900000)

ax.yaxis.set_major_locator(mticker.MultipleLocator(100000))

ax.yaxis.set_minor_locator(mticker.MultipleLocator(10000))

ax.legend(fontsize=tiny_labelsize, ncol=3, loc="upper left",

       handlelength=1, handletextpad=0.5, framealpha=1, columnspacing=0.5, labelspacing=0.25)

ax.tick_params(labelsize=labelsize)

​

fig.savefig(_FIG_FLDR + "overall_tweet_quantity.png",

         dpi=600, papertype='letter', orientation='portrait',

         facecolor='w', edgecolor='w', bbox_inches='tight')

​

fig = plt.figure(figsize=(16, 8))

ax = fig.add_subplot(111)

ax2 = fig.add_subplot(111)

tax = ax.twinx()

tax2 = ax.twinx()

​

tax.plot(_PD.index, _PD["COVID_VADER_smooth"].values,

     color=_COVID_COLOR, lw=5, zorder=2)

tax.plot(_PD.index, _PD["COVID_VADER_smooth_filled"].values,

      color=_COVID_COLOR, lw=5, linestyle="--", zorder=2)

​

ax.set_zorder(2)

ax.patch.set_visible(False)

ax.plot(_PD.index, _PD["CITY_VADER_smooth"].values, color=_CITY_COLOR, lw=5, zorder=3)

​

tax2.set_zorder(3)

tax2.patch.set_visible(False)

tax2.plot(_PD.index, _PD["COVID_VADER_avg"].values, marker="X",

       ms=ms, mec="k", color=_COVID_COLOR, lw=0, zorder=4)

tax2.scatter(_PD.index, _PD["COVID_VADER_CP"].values, s=2 * ms**2, marker="d",

          edgecolor=_COVID_COLOR, facecolor="w", zorder=5)

​

ax2.set_zorder(6)

ax2.patch.set_visible(False)

ax2.plot(_PD.index, _PD["CITY_VADER_avg"].values, color=_CITY_COLOR, marker="X", lw=0,

      ms=ms, mec="k", zorder=6)

ax2.scatter(_PD.index, _PD["CITY_VADER_CP"].values, s=2 * ms**2,

         marker="d", edgecolor=_CITY_COLOR, facecolor="w", zorder=7)

​

tax.fill_between([pd.Timestamp(_MISSING_DATE), pd.Timestamp(_MISSING_DATE) + _DAY], -1, 1,

              color=_ND_COLOR, alpha=0.5, zorder=1)

tax.fill_between([pd.Timestamp(_KOBE), pd.Timestamp(_KOBE) + _DAY], -1, 1,

              color=_KOBE_COLOR, alpha=0.5, zorder=1)

tax.fill_between([pd.Timestamp(_WHO), pd.Timestamp(_WHO) + _DAY], -1, 1,

              color=_WHO_COLOR, alpha=0.5, zorder=1)

tax.fill_between([pd.Timestamp("2020-03-16"), pd.Timestamp("2020-03-22")], -1, 1,

              color=_LD_COLOR, alpha=0.24, zorder=1)

tax.fill_between([pd.Timestamp("2020-03-22"), pd.Timestamp("2020-03-24")], -1, 1,

              color=_LD_COLOR, alpha=0.4, zorder=1)

tax.fill_between([pd.Timestamp("2020-03-24"), pd.Timestamp("2020-04-01")], -1, 1,

                 color=_LD_COLOR, alpha=0.84, zorder=1)

tax.fill_between([pd.Timestamp("2020-04-01"), pd.Timestamp(_END)], -1, 1,

              color=_LD_COLOR, alpha=1, zorder=1)

​

ax.set_title("Average VADER sentiment for both data sets", fontsize=labelsize)

​

ax.set_xlim(pd.Timestamp(_START_COVID), pd.Timestamp(_END))

ax.xaxis.set_major_locator(mdates.WeekdayLocator())

ax.xaxis.set_minor_locator(mdates.DayLocator())

ax.set_ylim(0.05, 0.2)

ax.set_ylabel(r"VADER sentiment Score (user-timeline data)", fontsize=labelsize, color=_CITY_COLOR)

ax.tick_params(axis='y', colors=_CITY_COLOR)

​

tax2.set_ylim(-0.2, 0.01)

tax2.yaxis.set_major_formatter(mticker.NullFormatter())

​

tax.set_ylim(-0.2, 0.01)

tax.set_ylabel(r"VADER sentiment Score (COVID-19 corpus)", fontsize=labelsize, color=_COVID_COLOR)

tax.tick_params(axis='y', colors=_COVID_COLOR)

​

ax2.set_ylim(0.05, 0.2)

​

for a in [ax, ax2, tax, tax2]:

a.set_xlim(pd.Timestamp(_START_COVID), pd.Timestamp(_END))

    a.xaxis.set_major_locator(mdates.WeekdayLocator())

    a.xaxis.set_minor_locator(mdates.DayLocator())

    a.xaxis.set_major_formatter(mdates.DateFormatter("%b %d"))

​

    a.yaxis.set_major_locator(mticker.MultipleLocator(0.05))

a.yaxis.set_minor_locator(mticker.MultipleLocator(0.005))

a.tick_params(labelsize=labelsize)

​

tax.plot([], [], color=_COVID_COLOR, lw=5, marker="X", ms=ms, mec="k", label=r"COVID-19 corpus")

tax.plot([], [], color=_CITY_COLOR, lw=5, marker="X", ms=ms, mec="k", label=r"user-timeline data")

tax.scatter([], [], marker="d", s=2 * ms ** 2, edgecolor="k", facecolor="w", label=r"Change-points")

tax.fill_between([], -1, 1, facecolor=_ND_COLOR, alpha=0.5, label=r"No data (COVID-19 corpus)")

tax.fill_between([], -1, 1, facecolor=_KOBE_COLOR, alpha=0.5, label=_KOBE_LABEL)

tax.fill_between([], -1, 1, facecolor=_WHO_COLOR, alpha=0.5, label=_WHO_LABEL)

tax.fill_between([], -1, 1, facecolor=_LD_COLOR, alpha=0.24, label=r"LD (NOLA)")

tax.fill_between([], -1, 1, facecolor=_LD_COLOR, alpha=0.40, label=r"LD (5 cities)")

tax.fill_between([], -1, 1, facecolor=_LD_COLOR, alpha=0.84, label=r"LD (16 cities)")

tax.fill_between([], -1, 1, facecolor=_LD_COLOR, alpha=1, label=r"LD (20 cities)")

​

tax.legend(fontsize=tiny_labelsize, ncol=5, loc="lower right",

        handlelength=1, handletextpad=0.5, framealpha=1, columnspacing=0.5, labelspacing=0.25)

​

fig.savefig(_FIG_FLDR + "VADER_average.png",

         dpi=600, papertype='letter', orientation='portrait',

         facecolor='w', edgecolor='w', bbox_inches='tight')

​

fig = plt.figure(figsize=(10, 4))

ax = fig.add_subplot(111)

​

ax.set_title("Distribution of VADER sentiment for COVID-19 corpus", fontsize=labelsize)

​

ax.step(_PD.index - pd.Timedelta(hours=12), _PD["COVID_VADER_perc_pos"].values,

     color="k", linestyle="-", where="post", lw=2, zorder=3)

ax.fill_between(_PD.index - pd.Timedelta(hours=12), _PD["COVID_VADER_perc_pos"].fillna(0),

             step="post", facecolor=_POS_COLOR, alpha=0.5, zorder=1, label="Positive")

ax.fill_between(_PD.index - pd.Timedelta(hours=12), _PD["COVID_VADER_perc_pos"].fillna(100), 100,

             step="post", facecolor=_NEG_COLOR, alpha=0.5, zorder=1, label="Negative")

​

ax.fill_between([pd.Timestamp(_MISSING_DATE), pd.Timestamp(_MISSING_DATE) + _DAY], 0, 100,

             facecolor=_ND_COLOR, alpha=0.8, zorder=2, label="No data")

ax.fill_between([pd.Timestamp(_WHO), pd.Timestamp(_WHO) + pd.Timedelta("1 day")], 0, 100, lw=0,

             color=_WHO_COLOR, alpha=0.8, label=_WHO_LABEL, zorder=2)

​

ax.set_xlim(pd.Timestamp(_START_COVID), pd.Timestamp(_END))

ax.xaxis.set_major_locator(mdates.WeekdayLocator(byweekday=1, interval=2))

ax.xaxis.set_minor_locator(mdates.DayLocator())

ax.xaxis.set_major_formatter(mdates.DateFormatter("%b %d"))

ax.set_ylabel(r"Percentage of tweets", fontsize=22)

ax.set_ylim(34, 54)

ax.yaxis.set_major_locator(mticker.MultipleLocator(5))

ax.yaxis.set_minor_locator(mticker.MultipleLocator(0.5))

ax.yaxis.set_major_formatter(mticker.PercentFormatter())

ax.legend(fontsize=tiny_labelsize, ncol=2, bbox_to_anchor=(0.35, 1.025), loc="upper center",

       handlelength=1, handletextpad=0.5, framealpha=1, columnspacing=0.5, labelspacing=0.25)

ax.tick_params(labelsize=labelsize)

​

fig.savefig(_FIG_FLDR + "VADER_pos_neg.png",

         dpi=600, papertype='letter', orientation='portrait',

         facecolor='w', edgecolor='w', bbox_inches='tight')
